# Genome Plasticity in Cultured *Leishmania donovani*: Comparison of Early and Late Passages

**DOI:** 10.3389/fmicb.2018.01279

**Published:** 2018-07-03

**Authors:** Roma Sinha, Mathu Malar C, Raghwan, Subhadeep Das, Sonali Das, Mohammad Shadab, Rukhsana Chowdhury, Sucheta Tripathy, Nahid Ali

**Affiliations:** ^1^Infectious Diseases and Immunology Division, CSIR-Indian Institute of Chemical Biology, Kolkata, India; ^2^Structural Biology and Bioinformatics Division, CSIR-Indian Institute of Chemical Biology, Kolkata, India; ^3^Academy of Scientific and Innovative Research (AcSIR), New Delhi, India

**Keywords:** *Leishmania donovani*, genomics, *in vitro* passage, promastigotes, genome plasticity

## Abstract

*Leishmania donovani* possesses a complex heteroxenic life cycle where infective metacyclic promastigotes are pre-adapted to infect their host and cope up with intracellular stress. Exploiting the similarities between cultured and sandfly derived promastigotes, we used early and late passage cultured promastigotes to show specific changes at genome level which compromise pathogen fitness reflected in gene expression and infection studies. The pathogen loses virulence mostly *via* transcriptional and translational regulations and long-time cultivation makes them struggle to convert to virulent metacyclics. At the genomic level very subtle plasticity was observed between the early and the late passages mostly in defense-related, nutrient acquisition and signal transduction genes. Chromosome Copy number variation is seen in the early and late passages involving several genes that may be playing a role in pathogenicity. Our study highlights the importance of ABC transporters and calpain like cysteine proteases in parasite virulence in cultured promastigotes. Interestingly, these proteins are emerging as important patho-adaptive factors in clinical isolates of *Leishmania*. We found that the currently available genome of *Leishmania* in the NCBI database are from late passages. Our early passage genome can act as a reference for future studies on virulent isolates of *Leishmania*. The annotated leads from this study can be used for virulence surveillance and therapeutic studies in the Indian subcontinent.

## Introduction

*Leishmania donovani* is an intracellular obligate parasite of mammalian macrophages and causes visceral leishmaniasis or *kala-azar*, which is a fatal disease if not treated on time. The disease severity varies from host to host, region to region as well as between parasite strains. Extensive research by different groups exploiting genomic, proteomic, metabolomic, immunologic, and animal models have pointed toward tripartite determinants mediating the development of this disease *viz*. the vector, host and pathogen. Although vector and host characteristics are important for symptomatic disease, parasite characteristics majorly determine the fate of the disease (McCall et al., 2013). Promastigotes are the culturable form of *Leishmania*. Stationary phase culture of *Leishmania* is expected to contain a large number of metacyclic parasites and have been routinely used for experimental infections. Metacyclic promastigotes are injected into the host during blood meal and are the infective stage of the parasite. We and others have observed that continuous axenic cultivation of *Leishmania* leads to loss of virulence over a period of time (Dey et al., 2002; Ali et al., 2013). The relative virulence of stationary phase promastigotes is proportional to peanut agglutinin negative promastigotes contained within these populations (da Silva and Sacks, 1987) which is attributable to exposed surface carbohydrates. It has also been observed that the *in vitro* maintenance of *Leishmania* promastigotes by cultivation over longer periods may reduce their ability to differentiate into amastigote forms (Moreira et al., 2012; Ali et al., 2013). Studies at the mRNA and proteomic levels indicated differential expression of virulence related genes in both promastigotes and amastigotes cultured axenically for long periods compared to freshly isolated/transformed or early passage parasites (Lei et al., 2010; Pescher et al., 2011; Ali et al., 2013; Magalhaes et al., 2014). These studies were done with the aim of standardizing protocols for the use of axenic *Leishmania* cultures in infection studies and to identify virulence factors. Studies at genomic level mostly identified parasite evolution in the Indian subcontinent under drug pressure or disease phenotype (Zhang et al., 2014; Imamura et al., 2016). As promastigotes are the infective form of *Leishmania*, and cultured stationary phase promastigotes show remarkable consistency with sandfly derived metacyclic promastigotes (Inbar et al., 2017), we chose the former system to unravel the genetic mechanism of parasite pre-adaptation to host environment and how it is lost with continuous *in vitro* passages. We report here the global changes in the genome and transcriptome of serially passaged *L. donovani* AG83 strain which cause specific alterations in few genes and their expression that lead to loss of virulence reflected in the infection studies. We also try to link these adaptive features with parasite’s nutritional environment and complex life cycle, and how closely they relate to the global evolution of *L. donovani* clinical isolates as available through published work. This study also throws light on the pathologically significant genes common to clinical and laboratory strains which must be considered for virulence surveillance at least in the Indian subcontinent.

## Materials and Methods

### Ethics Statement

All animal experiment protocols adhered to the guidelines of the Committee for the Purpose of Control and Supervision on Experimental Animals (CPCSEA), Ministry of Environment and Forest, Government of India, and were approved by the Animal Ethics Committee (147/1999/CPSCEA) of CSIR-IICB.

### Parasite Culture and Maintenance in Animals

*Leishmania donovani* (MHOM/IN/1983/AG83; ATCC repository number PRA^®^-413^TM^) amastigotes from infected hamster spleen were transformed to promastigotes in Schneider’s Drosophila medium (Sigma-Aldrich) at 22°C and sub-cultured as promastigotes in M 199 (Sigma-Aldrich) both supplemented with 100 U/ml penicillin, 100 μg/ml streptomycin, and 10% FCS (Sigma-Aldrich) (Banerjee et al., 2008). Parasites were sub-cultured till 25th passage for some experiments. Golden Syrian Hamsters, 5–6 weeks old, reared in institute facilities were used for the purpose of parasite maintenance.

### Macrophage Infection *in Vitro*

Macrophages isolated from the peritoneal cavity of hamsters (12–16 weeks old) by injecting chilled RPMI-1640-10% FCS were pooled and cultured overnight on glass cover slips as described elsewhere (Bhowmick et al., 2010). AG83 promastigotes of different passages were allowed to infect peritoneal macrophages (MoI of 10:1) for 3 h, washed with warm 20 mM PBS, pH 7.2 and further incubated in fresh medium till 72 h. At 24 and 72 h post-infection, cells were fixed in methanol and stained with Giemsa for microscopic determination of intracellular parasite numbers per 100 host cells. Data were analyzed using GraphPad Prism 5 software.

### Determination of Splenic and Hepatic Parasite Burden

Hamsters (5–6 weeks) were infected by injecting 2 × 10^7^ stationary phase promastigotes by intracardiac injection suspended in 20 mM PBS. Animals were sacrificed 8 weeks post-infection for determination of parasite burden by Leishman Donovan Units (LDU) in Giemsa stained impression smears and limiting dilution assay in fivefold serial dilutions of homogenized organs as described previously (Banerjee et al., 2008). Data analysis was performed on GraphPad Prism 5 software.

### High Throughput Genome Sequencing

High quality genomic DNA was extracted from stationary phase promastigotes of the early (HTI4) and late (HTI5) passage of *L. donovani* AG83 using a commercial procedure as recommended by the manufacturer (Qiagen, Germany). Size check, integrity and presence of contaminants in the DNA samples were assessed through gel electrophoresis. DNA purity was measured using a Nano Drop 2000 spectrophotometer (Thermo Scientific, Waltham, MA, United States). Two separate libraries of *L. donovani* AG83 were prepared one each for early and late passages. *De novo* paired end sequencing was done on Illumina HiSeq 2500 with the read length of 125 base pairs with an insert size of 250 bp. A total of 66 and 44 million raw reads were generated from early and late passages, respectively.

### Transcriptome Sequencing

For each passage, parasites were harvested by chilling on ice, spun down, and washed once in cold PBS solution, pH 7.4, and suspended in RNA Later. Total RNA was isolated from promastigotes of early (2nd), intermediate (11th), and late (25th) passage (referred to as q1, q2, and q3, respectively, in the figures) by using “Roche High Pure RNA Isolation Kit,” (Product no.11828665001) according to manufacturer’s protocol. The purity and concentration of each RNA sample was checked by using the Agilent 2100 bioanalyzer (Agilent Technologies, CA, United States) before proceeding for further downstream analyses. Paired end transcriptome sequencing was carried out on Illumina HiSeq platform, with the read length of 125 base pairs with an insert size of 250 bp generating 37–42 million raw reads (approximately). The library has been sequenced following manufacturer’s instructions using the HiSeq SBS Kit v4 (Part # 15034097Rev.B) to generate paired-end reads. Additional quality control of raw data using FastQC (11) was performed. The reads were preprocessed using Trimmomatic and the poor quality sequences, contaminated sequences were removed using the blast and blat based searches using the reference (LdBPK82A1; Bioproject: PRJNA171503) from Genbank. The high quality filtered reads were used for downstream analyses.

### Genome and Transcriptome Assembly and Functional Annotation

The early and late passage genomes were assembled using Allpaths-LG assembler (Ribeiro et al., 2012). We simulated reads with 6 K and 20 K insert sizes from the existing genomes of LdBPK282A1 retrieved from Genbank (PRJNA171503) with WGsim (Li et al., 2008).

The assembly from Allpaths was resolved up to chromosome level reference based genome assembly by nucleotide alignment using Nucmer and further using our in-house scripts^1^. RNAseq reads from all passages were aligned using blat (Kent, 2002) with the Genbank data of LdBPK282A1 for removal of contaminants. The cleaned reads were used for transcriptome assembly using Trinity transcriptome assembler (Grabherr et al., 2011; Haas et al., 2013) (Supplementary Table S1). We used BUSCO (Simão et al., 2015) for predicting the genome completeness and the core ortholog analysis was computed using eukaryotic genomes (Supplementary Table S2).

We predicted protein coding genes of the assembled genomes using Augustus (Stanke et al., 2008) and Scipio pipeline with LdBPK282A1 coding sequences as training material (Bioproject: PRJNA171503). Predicted genes were annotated using BLAST (Altschul et al., 2009) as well as InterProScan (Quevillon et al., 2005) searches. Whole genome protein sequences were submitted to the gene ontology (GO) and Kyoto Encyclopedia of Genes and Genomes (KEGG) analysis. GO ids were mapped back to their ontology functions using in house scripts. GO enrichment was analyzed in the categories of molecular function, cellular component and biological process. Metabolic pathways analysis was done using the KASS server (Moriya et al., 2007).

Additionally, we used companion server (Steinbiss et al., 2016) for predicting orthologous genes from the two culture passages using Genbank *Leishmania major* as the reference. This server predicted many interesting features including the pseudogenes present in the genomes that were not predicted by Augustus. Genomes of two passages were compared using MUMmer, NUCmer, and PROmer (Kurtz et al., 2004).

### Differential Expression Analysis of Transcripts

We carried out differential expression analysis of transcripts using Tuxedo suit tools (Langmead et al., 2009; Trapnell et al., 2012; Kim et al., 2013) by aligning the RNAseq reads of three passages with the reference genome of LdBPK282A1. The pattern of expression across samples was calculated by using the normalized Fragments per Kilo base of transcript per Million (FPKM) values. R scripts were used to construct box plots and perform hierarchical clustering (Supplementary Figure S1). Putative secretary functions were assigned to the DEGs by using Secretome tool (Bendtsen et al., 2004).

Total RNA was isolated from 4 × 10^7^ parasite promastigotes of different passages, using TriZOL-chloroform method. Pure RNA pellet was suspended in 50 μl DEPC treated RNase free water and concentration was measured using Nanodrop 2000 (Thermo Fisher Scientific). Almost 2 μg pure RNA was used for cDNA synthesis following manufacturer’s protocol using Revert Aid First Strand cDNA synthesis kit (Thermo Fisher, MA, United States). Differential expression analysis of parasite genes was performed using SYBR green master mix (Roche). Ld GAPDH was used as reference gene. Reaction was run in Roche light cycler 96 (Roche) and fold change of different genes was expressed as 2^-ΔΔ^*^C^*^t^, where Δ*C*t = gene *C*t- reference gene *C*t and Δ(Δ*C*t) = Δ*C*t test – Δ*C*t control. RNA was collected from *n* = 3 hamster and proceeded for further long passage preparation. Also data per group (A2, A11, and A25th passage) represents *n* = 3 experiments performed for each group.

### SNP Identification

Sequenced illumina genomic reads of early and late passages were aligned with the reference genome of LdBPK282A1 using Burrows-Wheeler Aligner (Li and Durbin, 2009), After marking the duplicates using PICARD tools the variants were called using the Genome Analysis toolkit (GATK) (Van der Auwera et al., 2013), obtained variants were filtered with the cutoff of QD < 2.0 ||MQ < 40||FS > 60.0|| ReadPosRankSum < -8.0.

### Copy Number Variant Detection

The reads mapped by using BWA were used to estimate the read depth for each sample at each chromosome using the coverage values from Samtools v0.1.18 (Li et al., 2009). After removing the duplicates using Picard the CNVnator (Abyzov et al., 2011) was used to identify copy number variants. We used 100 bp as the window size for creating the histogram bins of read depth in CNVnator. For CNVnator, we removed calls which were less than the cutoff of *t*-test calculated *e*-value of 1e-5.

### Cell Viability and Apoptotic Cell Death Assay

Promastigotes of early (2nd) and late (25th) passages were treated with graduated doses of miltefosine for 24 h and the percent viability was determined by enzymatic reduction of 3-[4,5-dimethylthiazole-2-yl]-2,5-diphenyltetrazolium bromide (MTT) to MTT-formazan. In parallel experiments the miltefosine treated promastigotes were analyzed for apoptotic cell death by AnnexinV-FITC and propidium iodide staining acquired in FITC and PE channels, respectively, on FACS LSR Fortessa and analyzed for percent FITC positive and FITC-PI double positive cells on FACS Diva software.

### Preparation of Leishmanial Antigen

The promastigotes (2nd–5th and 22nd–25th passage) were washed three times in cold 20 mM PBS, pH 7.2 and re-suspended at a concentration of 1.0 g cell pellet in 50 ml of cold 5 mMTris–HCl buffer, pH 7.6 and Leishmanial antigen (Lag) was prepared by ultrasonication as described earlier (Bhowmick et al., 2010). LAg was precipitated in 90% acetone-10% TCA, solubilised in rehydration buffer (7 M urea, 2 M thiourea, 2% Nonidet P-40, 2% dithiothreitol, and 2% pharmalyte, all from GE Healthcare LifeSciences, Little Chalfont, United Kingdom except NP-40 from Sigma) overnight and estimated by Bradford method (Bradford, 1976). Two independent batches were prepared from each passage for analysis.

### Isoelectric Focusing

The isoelectric focusing (IEF) was performed using the PROTEAN IEF Cell system (Bio-Rad). 500–600 μg of whole cell protein was added to 300 μl of rehydration buffer for 11 cm IPG strip (pH 4–7 non-linear, Bio-Rad). Protein sample was applied to the IPG strips and kept at room temperature (16–18 h) for passive rehydration. IEF was performed at 250 V for 20 min; 10,000 V for 2 h 50 min; 10,000–40,000 V/h and an optional step of 500–1000 V 10–15 h.

### Second Dimension-SDS-PAGE

Before the second dimension, proteins in the IEF strips were reduced in equilibration buffer (6 M urea, 2% SDS, 300 mM Tris–HCl pH 8.8, 20% glycerol) containing 20 mg/ml DTT and alkylated in dark with 25 mg/ml iodoacetamide, again dissolved in equilibration buffer, for 15–20 min each. Strips were washed with SDS-PAGE running buffer and separated across 12% SDS-PAGE gels (30% acrylamide, 0.8% bis-acrylamide) using a vertical system (Bio-Rad) and standard Tris/glycine/SDS buffer. Gels were run at 30 mA/gel until the tracking dye left the gel. The protein standard was purchased from Bio-Rad (pre-stained SDS-PAGE broad range). For western blot analysis, total cell lysates of early and late passage promastigotes were run on SDS-PAGE followed by incubation with antibodies for gp63 and HDAC. The images were developed using Luminata Forte (Thermo Fisher, MA, United States) on Gel Doc XR (Bio-Rad). The anti-gp63 antibody raised in rabbit (in house) and anti-mouse HDAC antibody (Cell Signaling Technology) were used for the assay.

### Spot Handling and Tryptic Digestion for Matrix-Assisted Laser Desorption/Ionization-Time of Flight Mass Spectrometry (MALDI-ToF MS/MS)

The 2-DE gels were stained with Coomassie Brilliant Blue G-250. Gel images were taken on the Gel Doc XR+ (Bio-Rad) and analysis was done using the PD Quest software version 8.0.1 (Bio-Rad Laboratories) and manual checking. The gel having a higher number of spots was used to locate the corresponding protein spots within different gels. In this study, we have applied a cut-off of at least 1.5-fold up-regulation/down-regulation of protein for the study. Experimental molecular weights and isoelectric points were calibrated according to Bio-Rad standard 2-DE PAGE marker. Spots were manually excised and processed according to the In-Gel Tryptic Digestion Kit (Thermo Fisher, MA, United States). The trypsin digested samples were concentrated using speed-vac before loading the samples for MALDI-TOF Mass Spectrometry analysis on 4800 MALDI ToF/ToF analyzer (Applied Biosystems, CA, United States). Zip Tip protein concentrator (Millipore, MA, United States) was used for concentrating and de-salting of the low abundance proteins. The samples were dissolved in a solvent consisting of 0.1% trifluoroacetate and 50% acetonitrile in MilliQ Water. Then, 0.5 μl of sample solution was mixed with 0.5 μl of matrix solution (1 mg/ml α-cyano-4-hydroxycinnamic acid dissolved in the aforementioned solvent), applied to a 384-MALDI sample target plate, and dried in air. Peptides were evaporated with a ND: YAG laser at 355 nm, using a delayed extraction approach. They were accelerated with 25 kV injection pulse for ToF analysis. Each spectrum was the cumulative average of 1000 laser shots. The MS/MS spectrum was collected in MS/MS 1 kV positive reflectron mode with fragments generated by post source decay. The MS/MS mass tolerance was set to ±20 ppm. After processing, 10 MS/MS precursors were selected (Minimum signal to noise ratio-50). Before each analysis, the instrument was calibrated with the Applied Biosystems 4700 Proteomics Analyzer Calibration Mixture. Data interpretation was performed using the GPS Explorer Software (Applied Biosystems, CA, United States), and an automated database search was performed using the MASCOT program (Matrix Science Ltd., London, United Kingdom).

### Data Availability

The Genome sequences of Early and Late passages of *L. donovani* MHOM/IN/1983/AG83 is available at NCBI with submission numbers GCA_001989955.1 and GCA_001989975.1, respectively.

## Results and Discussion

### Continuous *in Vitro* Passaging Leads to Loss of Virulence of Promastigotes

**FIGURE 1:**
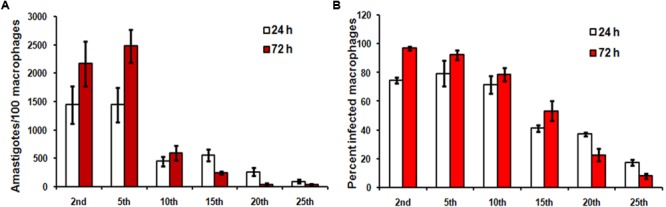
*In vitro* hamster derived macrophage infection with different passages. Hamster macrophages were infected with stationary promastigotes of *Leishmania donovani* AG83, as described in Section “Materials and Methods” and the cells were incubated for 24 h and 72 h at 37°C, 5% CO_2_. **(A)** The number of amastigotes in each passage and **(B)** the percentage of infected cells were analyzed by counting 100 cells. Data shown are mean ± SE of experiment performed in quadruplet.

**FIGURE 2:**
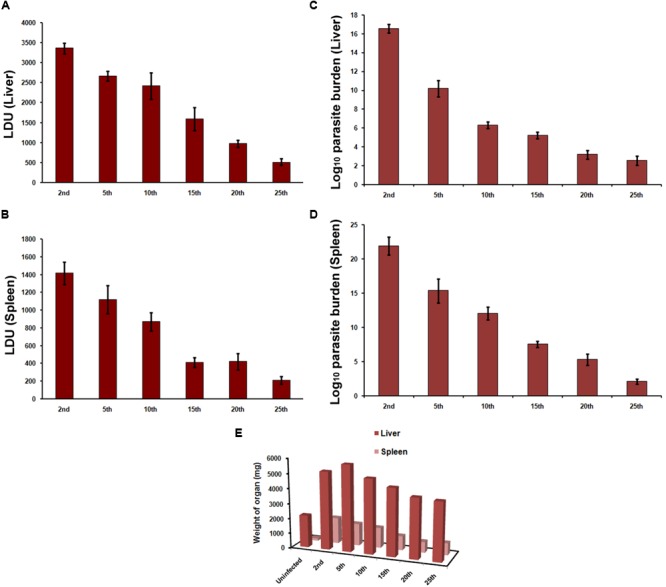
Infection in hamsters. Hamsters were infected by intracardiac injection of 2 × 10^7^ *L. donovani* AG83 promastigotes. Animals were sacrificed at 8 week post-infection and **(A,C)** liver and **(B,D)** spleen parasite burden was determined by **(A,B)** LDU and **(C,D)** LDA. Data represent mean ± SE of two independent experiments (*n* = 4–6). **(E)** The mean weights of liver and spleen were compared with respective organ weights of uninfected animals.

We first compared the infection causing capacity of promastigotes from different passages in *vitro* and *in vivo*. Stationary promastigotes recovered from different passages were quantified and employed in the experiments. Hamsters and hamster derived macrophages were highly susceptible to infection, with infection persisting up to 25th passage (**Figures 1, 2**) and displayed a decline in infectivity with more time spent in the culture. The average number of amastigotes per macrophage was 14.4 ± 3.2 at 24 h, which increased to 21.6 ± 3.9 at 72 h (**Figure 1A**) post-infection with early passage. There was over a 50-fold reduction in the number of intra-macrophagic amastigotes compared to the 2nd passage when parasites of 25th passage were used for infection (**Figure 1**, *p* < 0.0001, One way Anova). Parasites from the 2nd passage could infect 96.83 ± 1.4% of macrophages at 72 h while only 8 ± 1.6% cells were infected with promastigotes from 25th passage (**Figure 1B**). From these experiments it seemed the infectivity is affected at two stages, first at the level of host-parasite interaction evident from lower infection at 24 h, and second, parasite sustenance inside macrophages, as seen in clearance of parasites after 72 h. Hamsters infected with 25th passage promastigotes presented over 6.5-fold less parasite burden in both liver (LDU of 515.6 ± 82.44) and spleen (LDU of 211.5 ± 42.21) as compared to those infected with the 2nd passage (LDU of 3358 ± 132.9 and 1416 ± 126.6, respectively, in liver and spleen) (**Figures 2A,B**, *p* < 0.0001, One Way Anova) at 8 week post-infection. As the number of live amastigotes inside the organs gives an idea of the disease severity, we additionally assessed the parasite burden in the infected liver and spleen of differentially infected hamsters by serial dilution method. The number of parasites showed a gradual decline with passage number thus reinstating the fact that continuous axenic culture of promastigotes leads to loss of virulence of the parasite. As shown in **Figure 2C**, the number of live amastigotes came down from 10^16^ in the liver of hamsters infected with virulent parasites (2nd passage) to as low as 100 parasites in the animals with attenuated infection (25th passage) (*p* < 0.0001, One Way Anova). The splenic infection level was very high in hamsters infected with early passage promastigotes, and reached levels as high as 10^21^ (**Figure 2D**, *p* < 0.0001, One Way Anova) whereas the parasites failed to survive and multiply in the animals infected with late passage, and only about 100 parasites could be detected in the piece of organ examined by us. The hamsters with mild infection also demonstrated reduced liver and spleen size (**Figure 2E**) unlike the severely infected groups. Thus early passage promastigotes displayed increased fitness inside host cells reflected in higher parasite burden while late passage promastigotes were successfully cleared by macrophages.

### Late Passage Genome Has Higher Number of Pseudogenes

**FIGURE 3:**
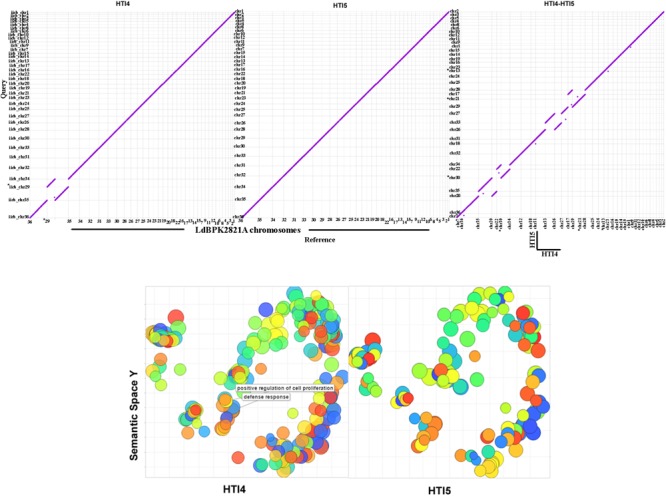
Mummerplot comparisons and Gene Ontology analysis of early and late passage genomes to the NCBI reference genomes. **(Upper)** The *y*-axis represents HTI4 (left) or HTI5 (middle) whole genome assemblies compared to *x*-axis representingLdBPK282A1 assembly. The HTI4 (*x*-axis) versus HTI5 (*y*-axis) chromosomal assembly comparison is given in the right panel. The dots represent the positions of conserved DNA sequences on the genomes. **(Lower)** REVIGO was used to visualize the summary of significantly enriched GO terms. The scatterplots show the cluster representatives in a two dimensional space derived by applying multidimensional scaling to a matrix of the GO terms’ semantic similarities for early (left panel) and late (right panel) passages. Clustal differences are represented in boxes. Bubble color indicates the user-provided *p*-value; size indicates the frequency of the GO term in the underlying GOA database (bubbles of more general terms are larger).

Genome plasticity in *Leishmania* has been linked to its ability to adapt to different environments (Sterkers et al., 2012). This prompted us to check for the genetic adaptations which may account for adaptability to growth in culture medium and the associated loss of infectivity as a result of continuous passaging. The genomes of early and late passages were assembled into complete 36 chromosomes with a genome size of 32.2 and 32.1 MB, respectively (**Table 1A**). The genomes of these two passages, however, had about 11.3% gaps compared to 3.67% gaps in LdBPK282A1 from Genbank [Accession number: GCA_000227135.2]. A chromosome wise comparison of early and late passages with LdBPK282A1 (Supplementary Figure S2) didn’t reveal much difference, whereas the whole genome comparisons clearly pointed out the small but prominent changes in the genome of the late passage, which was comparable to the NCBI reference LdBPK282A1 strain (**Figure 3**, upper panel). We used RATT and companion for transferring annotation from LdBPK282A1 strain (Downing et al., 2011) that resulted in 7563 and 7552 predicted protein coding genes, respectively, in early and late passages, compared to 7967 protein coding genes in LdBPK282A1 (**Table 1B**). The less number of protein coding genes from our cultures are mostly attributed to the presence of assembly gaps. The numbers of predicted pseudogenes in early and late passages were 73 and 82, respectively, and this number is significantly higher than 54 pseudogenes predicted in LdBPK282A1 strain, but similar to what is reported in the Srilankan *L. donovani* (70) strains (Zhang et al., 2014). Presence of large protein families such as calpain-like proteases and amastins are found to occur in large directional clusters in Genbank strain as well as our strains. We noticed a large amastin cluster in chromosome 8 in Genbank that are present in chromosome 10 in our strain (**Table 2**).

**Table 1A:** Summary of assembly statistics for early passage (HTI4), late passage (HTI5) and Genbank *Leishmania donovani* LdBPK282A1 genomes.

Assembly name	Number of contigs	Number of chromosomes	Total assembly size (in bases)	Largest chromosome (in bases)	Smallest chromosome (in bases)	Gaps (in bases)	N50 and GC%
HTI4	2382	36	32196393	2743999	284264	3663498	1058081, 59%
HTI5	2445	36	32148377	2714535	283355	3653324	1058043, 58%
LdBPK282A1 (GCA_000227135.2)	2152	36	32444968	2713248	283432	1192833	1024085, 59%

**Table 1B:** Gene prediction statistics for early (HTI4) and late (HTI5) passages using companion server.

Genome name	Gene	Protein coding	ncRNA	Pseudo	snRNA	SnoRNA	tRNA
HTI-4 Early passage (2nd)	7656	7563	14	73	2	1	67
HTI-5 Late passage (25th)	7643	7552	15	82	2	1	66
LdBPK282A1 (GCA_000227135.2)	8079	7967	37	54	0	0	64

**Table 2:** Gene Copy number in early, late passages and Genbank strain LdBPK282A1.

Gene product	Family size			Distribution on chromosomes		
	HTI4	HTI5	LdBPK282A1	HTI4	HTI5	LDBPK282A1
Kinesins	51	50	49	Scattered	Scattered^∗^	Scattered^∗^
Protein kinases	259	258	255	Scattered	Scattered^∗^	Scattered^∗^
MAP kinases	17	17	19	Scattered^∗^	Scattered^∗^	Scattered
Amastins	14	15	26	10,24,28,30,34,36	10,24,28,30,34,36	8,24,28,29,30,34
PSA2 (GP46) metalloproteases	28	29	29	Scattered^∗^	Scattered	Scattered
Serine peptidases	17	18	13	Scattered	Scattered	Scattered
Protein phosphatase	120	118	86	Scattered	Scattered	Scattered
Tuzins	4	4	6	8, 29^∗^,34	8, 29^∗^,34	8,29,34
Amino acid permeases	15	15	18	Scattered^∗^	Scattered^∗^	Scattered
HSP	11	12	10	Scattered^∗^	Scattered^∗^	Scattered
Calpain-like cysteine peptidase	29	31	26	4,17,18,20(7),21,25, 27(5),31(6),32,33,36	4,17,18,20(8),21,25, 27(4),31(7),32,33,34, 36	4,14,18,20(8),21, 25,27,30,31(5),32,33,34
Phosphoglycan β 1,3 galactosyltransferases	3	4	10	2^∗^,14,31^∗^,36^∗^#	2^∗^,14,31^∗^,36^∗^#	2,14,31,36
Dynein heavy and light chain	44	44	44	Scattered	Scattered	Scattered
Helicases	84	84	72	Scattered	Scattered	Scattered
Pteridine transporters	1	1	2	6,10^∗^#	6,10^∗^#	6,10
Microtubule-associated proteins	72	72	72	Scattered	Scattered	Scattered
ABC transporters	39	42	39	Scattered^∗^	Scattered	Scattered^∗^
Vesicle transporters	4	4	4	11,23,31,32	11.23,31,32	11,23,31,32
DNAJ protein/chaperone	61	61	29^∗∗^	Scattered	Scattered	Scattered^∗∗^
Long-chain fatty acid CoA ligases	9	9	9	1,3,13,19^∗^,28,36^∗^#	1,3,13,19^∗^,28,36^∗^#	1,3^∗∗^,13,19^∗^,28,36^∗^#
Cyclophilins	1	15	13^∗∗^	1,6,16,18,22,23,24,25, 30,31,33,35,36	1,6,16,18,22,23,24,25, 30,31,33,35,36	1,6,16,22,23,25,30,31, 33,35,36
Histone acetyl transferase/histone deacetylase	8	8	7^∗∗^	8,14,16,21,24,26,28	8,14,16,21,24,26,28	8,14,16,21,24,28
Nucleoside hydrolase	4	4	3^∗∗^	14,18,26,29	14,18,26,29	14,18,29

*^∗^One or more genes not present due to assembly gaps. ^∗∗^Gene prediction error. ^#^Gene absent in the scaffold due to assembly gaps.*

The GO Biological Process annotation of early and late passages indicates there are no changes in the gene numbers of processes in early and late passages. However, genes responsible for defense system (GO: 0006952) and positive regulation of cell proliferation (GO: 0008284) are missing in later passages (**Figure 3**, lower panel and Supplementary Data Sheet S1). Thus repeated *ex* host passages have made the late passage parasites suited to extracellular life where selection pressure against host defense system is not needed. Further, the cellular machinery needed for transformation to infective form is also perturbed which may impair their ability to infect the host.

### Chromosome Copy Number Variation Drives Gene Expression in *Leishmania*

The previous studies on *L. donovani* (Leprohon et al., 2009; Dumetz et al., 2017) reported that aneuploidy and Copy number variants regulate the gene expression. In our study we identified SNPs from early and late passages. Total 4390 heterozygous loci were found in early passage and 4356 heterozygous loci were found in late passage of the genome.

The local copy number variant detection was performed on the basis of read depth binning ratio approach. It was already known that deletion and duplication event plays an important role in these genomes to maintain the virulence in the different environmental conditions (Dumetz et al., 2017).

The copy Number variation on our passages was done using LdBPK282A1 as reference, with an *e*-value cut-off of 1e-5 for filtering. Three hundred and eighty CNV events were qualified in late passage and 365 CNV events were qualified in early passage (Supplementary Data Sheet S2). The size of CNV events ranged from 0.7 to 271 kb. We checked for the regions which were overlapping with the protein coding genes. A total of 230 deletion events and 135 duplication events were identified in the early passage while 234 deletions events and 146 duplication events were identified in the late passage genome (Supplementary Data Sheet S2). Uniq gene lists that had undergone changes are listed in Supplementary Data Sheet S2: uniq_CNV. The genes undergoing structural changes in later passages compared to earlier passages comprised of several ABC Transporters (10), Amino acid transporters (7), Amastins (3), GP63, calpain like cysteine proteases to name a few. Interestingly, we have also reported changes in gene expression profiling of GP63 and Calpain like cysteine proteases between the late and early passages. We found more CNV events in chromosomes 5, 6, 8, 15, and 31 in the genome of early passage as per earlier reports (Laffitte et al., 2016; Iantorno et al., 2017). High number of CNV events play a major part in the gene expression regulation of *in vitro* cultured promastigotes (Dumetz et al., 2017). Interestingly we observed an increased frequency of CNVs in the protein coding regions of the late passage particularly in chromosome 3, 4, 13, 16, and 20. Protein coding regions corresponding to calpain like cysteine protease genes on chromosome 20 displayed multiple duplication events. Phosphoglycerate kinase B, cytosolic fragment on chromosome 20 showed an insertion event in the late passage which was not found in the earlier passage. This gene was reported to be over expressed in Srilankan *L. donovani* strains causing visceral disease versus those causing cutaneous lesions (Zhang et al., 2014). Noticeable protein coding genes falling in the variant regions on chromosome 13 of late passage genome were some acetyl transferases including histone acetyltrasferase, *N*-acetyl transferase subunit ARD1, RAS-related protein RAB5, mitogen activated protein kinase 2, etc. which may have a role in cell cycle progression and morphogenesis of *Leishmania* (Wiese, 1998; Yadav et al., 2016).

### Single Nucleotide Polymorphisms in the ABC Transporter Coding Genes

**FIGURE 4:**
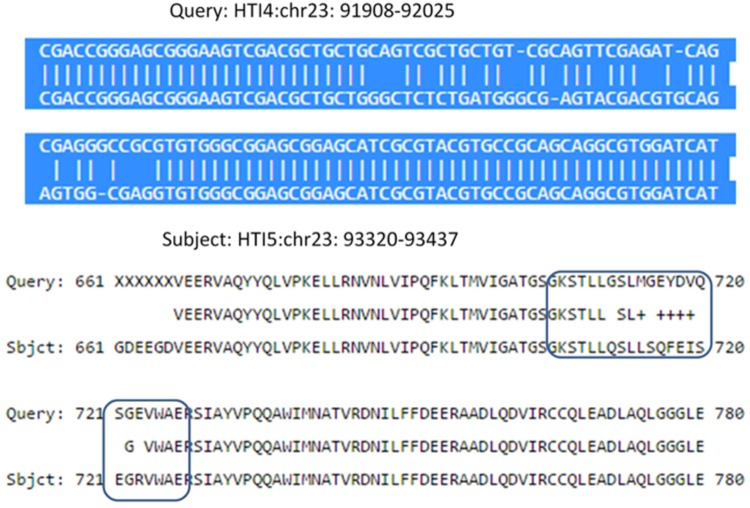
Nucleotide and protein sequence comparison in *L. donovani* ABC transporter gene in Chr 23 of early and late passages. **(Upper)** Shows multiple nucleotide polymorphisms in HTI4 and HTI5 including indels and nucleotide substitutions. **(Lower)** Describes the protein sequence polymorphism as a result of sequence changes. The differences leading to functional loss are marked as boxes.

The evolution of pathogenicity in microbes has been attributed to ordered changes in the functionality of the genes as a result of physiological constraints encountered by the organism in their immediate environment. A KEGG pathway analysis didn’t show major differences in the number of genes involved (Supplementary Data Sheet S3) although detailed mutational analysis revealed interesting changes linked directly or indirectly to loss of virulence in the later passage. Among the defense gene products, ABC transporters are important proteins involved in drug resistance, nutrient acquisition and pathogen virulence (Glavinas et al., 2004) and have expanded in the pathogenic protists as a parasitic adaptation (Zhang et al., 2014; Jackson et al., 2016). Genomes of *L. donovani* AG83 in early and late passages contain 42 copies of the genes spread over the genome. However, there are at least two instances where the ABC transporters are undergoing polymorphism that could result in reduced pathogen fitness. ABC transporter at LDON_230007700.1; HTI5:chr23: 91219–96174 in late passage has undergone several changes at the nucleotide level leading to an inactive ABC transporter in the late passages. Our Copy Number variant analysis also detected CNV duplication event of 12000 bp in the region of 86801–98800 in chromosome 23 in late passage. In earlier passages, a functional ABC transporter [HTI4:Chr23:91908–92025] resided inside a larger gene locus at HTI4:chr23:89807–101617 (**Figure 4**). We found a CNV duplication event as well in chromosome 23: 86801–98700 in early passage that corroborates this finding.

**FIGURE 5:**
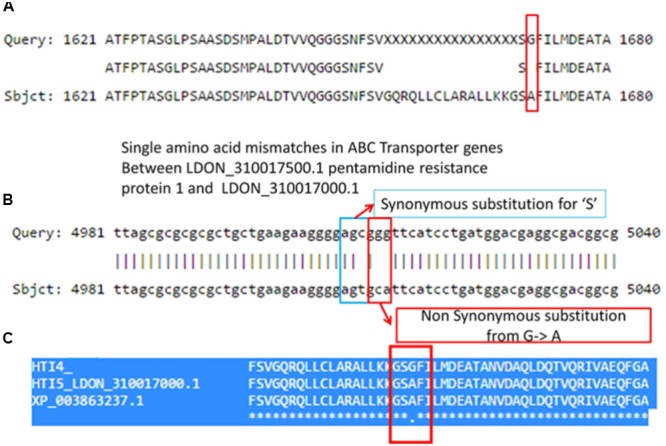
Synonymous and non-synonymous substitution in early and late passages leading to altered ABC transporter genes in chromosome 31. **(A)** A pair wise comparison between the protein coding genes in HTI4 and HTI5 showing single amino acid mismatches. **(B)** Nucleotide comparison in two genes indicates few substitutions, out of which the first one is a synonymous substitution and the second one is a non-synonymous substitution. A comparison between HTI4, HTI5 and Genbank strain **(C)** clearly indicates change of G–A in late passage same as the reference genome.

**FIGURE 6:**
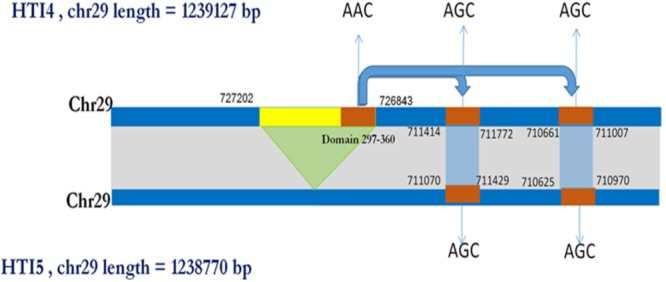
Schematic showing domain copy of MFS gene in Chromosome 29. A pseudo gene of MFS class of proteins present in HTI4 is missing in HTI5. However, a small domain from region 297–360 is being copied at two different locations in chromosome 29 with a non-synonymous substitution (AAC->AGC).

Interestingly, there is another ABC transporter gene in chromosome 31 with gene id LDON_310017500.1 in HTI4 and gene id LDON_310017000.1 in HTI5 where a substitution at 5012th position (GGG ->GCA a non-synonymous substitution which leads to a change from G->A amino acid at 1671st position in HTI5 (**Figure 5A**). The same gene undergoes two substitutions at the 5010th position (from AGC ->AGT) (**Figure 5B**) leading to synonymous substitution. The Genbank *L. donovani* strain LdBPK282A1 gene corresponding to this gene has the ‘A’ variant in this locus (**Figure 5C**). This observation is also supported by our CNV analysis in chromosome 31 as well as by Imamura et al. (2016). This gene codes for pentamidine resistance transporter protein. Major facilitator Protein (MFS class) is responsible for solute transport *via* membrane. Recently their role in stress responses and virulence has been proposed by various groups (Shah et al., 2014; Zhang et al., 2014). One such gene in HTI4 has been pseudogenized and this gene is absent in HTI5 (assembly gaps can’t be ruled out as one of the plausible causes though). A small segment of the gene (297–360 bp) is copied at two places both in HTI5 and HTI4 with one point non-synonymous mutation (AAC->AGC) (gene id: LDON_290021200 in HTI4) resulting in N ->S (**Figure 6**). The presence of this duplicated domain and its role in gene regulation and pathogenicity may be intriguing. Functional members of this family is present in duplicates in HTI5 as well as HTI4 (HTI5: Chr29: 711070–711429; HTI5: Chr29: 710625–710970 and HTI4: chr29: 711414–711772; HTI4:chr29: 710661–711007). This indicates selection pressure is working on MFS classes of proteins. Studies on pathogenic organisms demonstrate that the acquisition of iron is very important in the intracellular pathogenesis process and *Leishmania* possesses molecular machinery for iron regulation (Huynh and Andrews, 2008). Interestingly, these transporter proteins have links to the MFS superfamily (Huynh et al., 2006) and insight into genetic structure, mechanism and regulation via domain duplication in this class of proteins appears intriguing in designing targeted therapies.

**FIGURE 7:**
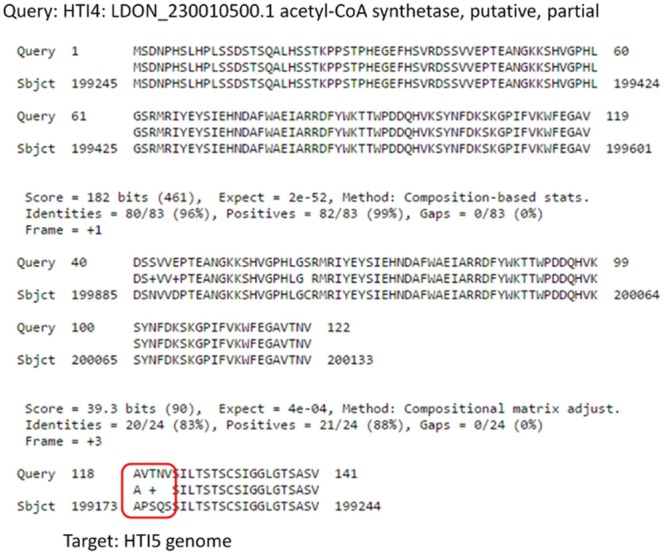
Comparison of Acetyl Co-A synthetase genes in early and late passage. Gene coding for Acetyl CoA Synthetase undergoes several substitutions in the late passage which makes it non-functional.

A fully functional acetyl-CoA synthatase gene in HTI4 has undergone modification [chr23: 199275–199598 in HTI5 and chr23: 198391–19851 in HTI4] leading to loss of function (**Figure 7**). There is also domain duplication in HTI4: LDON_230010500.1 from 1 to 118th position. The stringency in nutrient acquisition is key to preparedness of the parasite to intracellular environment (Carman et al., 2008) and loss of function of acetyl-CoA synthetase may restrict the parasite’s capacity to thrive in nutrient poor conditions using alternative carbon sources (McConville et al., 2015). Domain duplication in the early passage promastigotes (HTI4) indicate enhanced intracellular survival strategy.

**FIGURE 8:**
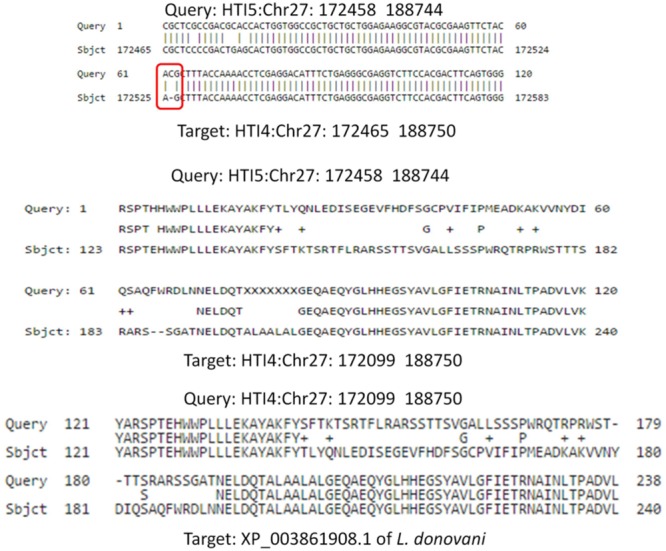
Frameshift mutation in Calpain like cysteine protease gene due to a single insertion in late passage. A single insertion in HTI5 (as well as Genbank LDBPK 282 A1) completely changes the coding frame of the gene (Upper panel) making it inactive. The changes in late passage and Genbank strain is consistent.

Calpain like proteases play a very important role in infection process. A single insertion in 172519th position in HTI5 on chromosome 27 (Chr27: 172458–188744) leads to frameshift mutation causing this gene to become a pseudogene. The corresponding functional gene in early passage is present at Chr27: 172465–188750 (**Figure 8**). In case of Genbank *L. donovani* genome, the protein sequence is more identical to the HTI5 protein sequence. Recent reports have pointed toward the regulatory role played by expressed pseudogenes in cancer cells and parasites (Wen et al., 2011). To check the downstream impact of mutations in ABC transporters and CALPs, we checked the relative sensitivity of early and late passage promastigotes to the drug miltefosine. Early passage parasites displayed an IC_50_ of 30 μM whereas the late passage promastigotes showed increased apoptotic death under drug pressure and an IC_50_ of 18 μM (Supplementary Figure S3). Interestingly, parasite CALPs may serve other functions in the intracellular form which determine disease outcome and host responses (Branquinha et al., 2013) and are thus potent drug targets. This may open up new avenues in understanding *Leishmania* biology. The implications of these modifications need further investigation.

### Comparative Transcriptome Profiles Between Passages Reveal Calpain Proteins Play an Important Role in Maintaining Virulence

**FIGURE 9:**
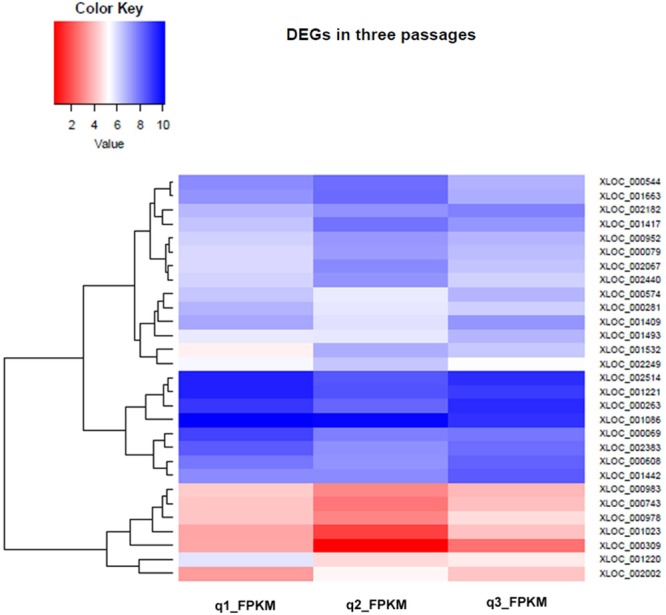
Hierarchical Clustering Heatmap of differentially expressed genes in early, intermediate and late passages. Heat maps were generated to show the comparative log 2 abundance ratios between early, intermediate, and late passages using RNAseq data. Upregulated proteins are depicted by blue bars, downregulated proteins by red bars.

The information from genomic studies done in this work as well as previous studies on axenic promastigotes indicated a strong link between parasite pre-adaptation and virulence. We hypothesized that continuous axenic cultivation may lead to altered transcript expression. To check for specific changes we analyzed the differential levels of transcript expression in three different culture stages (early, intermediate, and late passages) using RNAseq. Overall, there was no massive transcript expression switch between the early and late passages (**Figure 9** and Supplementary Figure S1) although specific genes presented differential expression (Supplementary Data Sheet S4) which correlated to certain extent with the CNV. The relative gene expression of a few genes was validated by RT-PCR (Supplementary Figure S4). Among the genes coding for surface active proteins, there was down regulation in membrane bound acid phosphatase and surface antigen like protein transcript levels (Supplementary Data Sheet S4) in the later passages. Acid phosphatase activity is needed for virulence and is also involved in endosome sorting (Katakura and Kobayashi, 1988). This may also be an adaptive response to the hydrolytic environment encountered inside sandfly gut and mammalian cells (Papadaki et al., 2015). Cyclin-dependent kinase pho85-like protein is a morphogenesis related protein having strong link to virulence in *Ustilago* (Castillo-Lluva et al., 2007) although its function in *Leishmania* has not been elucidated to date; it is implicated in environmental signaling in yeast (Carroll and O’Shea, 2002). This gene was under-expressed in the later passages. Among the cytoskeletal and flagellar proteins, a paraflagellar rod protein 1D (PFR 1D) was over expressed in early passage compared to the intermediate passage consistent with previous proteomic data (Magalhaes et al., 2014) on differentially passaged promastigotes. PFR 1D is essential for proper flagellar motility, a marker of promastigote virulence (Ginger et al., 2008) and it seems that flagellar morphology gets affected after few passages and ultimately leads to loss of virulence. Interestingly, multiple members of calpain like cysteine proteases (CALPs) (XLOC_001409, XLOC_000608, XLOC_001023; Supplementary Data Sheet 4) presented a typical pattern of expression where they were down-regulated between early and intermediate passages and ultimately had increased expression in the final passages. Some of the calpains have lost protease domain to gain microtubule organization function like SMP-1 (Branquinha et al., 2013). SMP-1 is required in all promastigote stages and also for transformation of amastigotes to promastigotes for the development of flagella (Tull et al., 2010). Thus over expression of these atypical CALPs in the later passages indicate struggle of the promastigotes to convert to infective forms. The role of calpains in signal transduction and cytoskeletal remodeling is well documented in trypanosomatids (Galetovic et al., 2011), and structural deformities may affect the virulence function of the parasite.

Metabolic reprogramming is a hallmark of *Leishmania* life-cycle where the parasite switches between two extreme environmental conditions. In the sandfly gut glucose and amino acids are the primary source of carbon while once inside the host, fatty acids and amino acids provide the necessary carbon for metabolism. Increased transcript abundance of 3-hydroxyisobutyryl-coenzyme a hydrolase-like protein in the early passages relative to the late is in consistency with the preparedness of the infection ready parasites to use beta-oxidation of fatty acids, as observed by others (Alcolea et al., 2009) (Supplementary Data Sheet S4). As opposed to the earlier observations (Moreno et al., 2014), level of tyrosine amino transferase presented a reverse trend, as it was slightly downregulated in the early passage, although protein expression may reveal different results as observed previously for *Trypanosoma cruzi* (dos Santos et al., 2012). Purine acquisition and inter-conversion is an important determinant of environmental sensing and morphogenesis in *Leishmania*. We observed a decrease in expression of a putative ribonucleoside-diphosphate reductase small chain, involved in this pathway in the late passage promastigotes in sync with earlier observation in *L. donovani* under purine stressed condition (Martin et al., 2014). On the other hand an ATP diphosphohydrolase was upregulated in the virulent parasites indicating preparedness for host-cell invasion and immunomodulation (Figueiredo et al., 2016) reflected in the differential host response in our infection studies. Amino acid transporter (aATP11) which has also been associated with purine starved condition (Martin et al., 2014) triggering parasite differentiation to metacyclic form (Inbar et al., 2017) was upregulated in the late passage, again reinstating the fact that these parasites lag in morphological transformation and try hard to attain infective status when growing in culture for a long time. Prostaglandin f2-alpha synthase involved in prostaglandin biosynthetic pathway is reduced in expression in the more infective promastigotes while the gene expression is more pronounced in the later passages indicating non-completion of metacyclogenesis as reported earlier (Araujo-Santos et al., 2014). *Leishmania* salvage folate and pteridines from their host or vector as they cannot synthesize them. The expression of these genes is higher in infective promastigotes and is known to aid in survival under extreme intra macrophagic conditions (Papadopoulou et al., 2002; Alcolea et al., 2010). As expected a gene for this protein on chromosome 6 was upregulated till 11th passage followed by a significant under expression in the late passage (**Figure 9** and Supplementary Data Sheet S4). Altogether it signifies that nutrient availability is strongly linked to gene expression in *Leishmania* and that many metabolites and classical metabolic enzymes are capable of altering the state of cellular differentiation and development as has been extensively studied in cancer cells (van der Knaap and Verrijzer, 2016). Metallopeptidases are important virulence factors in *Leishmania* and we already observed differential regulation of the major surface peptidase gp63 at protein level. Here, we observed slightly upregulated expression of two peptidases a metallopeptidase and a thimet oligopeptidase in the late passage. Transcriptomic data from late logarithmic phase promastigotes of *Trypanosoma brucei* growing in normal and purine supplemented medium displayed under expression of thimet oligopeptidase A in the former system linking it to purine stressed condition when approaching metacyclic state (Fernandez-Moya et al., 2014) corroborating our finding.

As pointed out above, genetic and epigenetic control of gene expression is central to adaptation of the parasite to its immediate and prospective environment. Consistent with that we observed an increased abundance of a histone-like transcription factor (CBF/NF-Y) and archaeal histone transcript in early passage relative to the late passage. The histone modifying enzyme histone deacetylase was also over expressed in the early passage. This protein has been associated with the disease causing stage of the parasite (Vergnes et al., 2005) and its higher activity may be linked to more invasiveness in the parasite. The higher protein level expression of HDAC in early passage promastigotes was also confirmed by western blot on whole cell lysates (Supplementary Figure S5). Together with this, protein modifying enzyme farnesyltransferase presented a downward trend in the late passage (**Figure 9** and Supplementary Data Sheet S4). This gene product has been associated with severe growth impairment in trypanosomatids (Gillespie et al., 2007). A putative pre-mRNA splicing factor ATP-dependent RNA helicase, member of DEAD box RNA helicase family presented increased expression till 11th passage. These proteins were shown to be strongly associated with mRNA of differentiating promastigotes as well as interactome of *in vitro* cultured amastigotes (Saxena et al., 2007). These proteins are mostly transiently upregulated or downregulated controlling the translational machinery. One of them has been associated with growth defects in *L. infantum* (Barhoumi et al., 2006). DNA repair related MutS-like proteins play important role in preventing genotoxicity and oxidative damage, a survival strategy adapted by many prokaryotic and eukaryotic pathogens (Genois et al., 2014), was upregulated in the virulent passage. Among the other differentially expressed transcripts are non-protein coding rRNA genes and conserved proteins with unknown functions. Non-coding RNAs along with proteins in the secreted exosomal milieu of *Leishmania* are known to modulate host–parasite interaction and changes in the composition of these exosomes may affect infectivity (Lambertz et al., 2015).

The results indicate that subtle genomic and transcriptomic variations drive the adaptation of promastigotes to culture condition and these changes mostly lead to loss of virulence in addition to morphological and metabolic modifications. Of particular interest is the role played by peptidases of different classes mainly metallopeptidases and calpain-like cysteine proteases in host–parasite interaction. Another important aspect is the importance of purine stress and modes of purine acquisition which modulate parasite and host responses to infection.

### Significant Regulation of Virulence Proteins in Comparative Protein Expression Studies

Till date the loss of infectivity in late passage culture has been explained by reduced expression of virulence factors, particularly those which can interact with and modulate the host cells. Proteomic comparison of crude membrane extracts of *L. donovani* AG83 promastigotes (LAg) revealed altered protein expression, particularly a decrease in expression of some known virulence factors after continuous passaging (**Table 3** and Supplementary Figure S6). There was very little overlap between transcriptomic and proteomic data which reinstates the fact that leishmanial gene expression is majorly controlled at post-transcriptional level (Lahav et al., 2011). A total of 35 differentially expressed spots were identified, out of which 31 spots presented higher expression in the early passage while four spots were highly expressed in the late passage. For example, gp63 (Olivier et al., 2012), HSP 70 (Ramirez et al., 2013a), elongation factor 1 alpha (Nandan et al., 2003), etc. have all been reported as virulence factors and were over expressed in the early passage. Western blot analysis on whole cell lysates of early and late passage promastigotes confirmed increased expression of gp63 in the virulent parasites (Supplementary Figure S5). The pyruvate dehydrogenase complex and its component dihydrolipoamide acetyltransferase has been associated with multiple functions including oxidative defense and regulation of gene expression in various pathogenic microbes (Spalding and Prigge, 2010), and presented an increased expression in the early passage. Apart from these, many hypothetical proteins also displayed differential protein expression and may determine the invasive capacity of the parasite. Transcript expression of each gene is also presented in **Table 3** and presented a similar trend. Most of the proteins which displayed differential expression in our study form a part of the infectious exosomal proteins (Silverman et al., 2010). The changes in exosomal milieu probably changes the modulatory capacity of the parasites of late passage inside macrophages, which in turn make them more susceptible to microbicidal attack. The ultimate protein repertoire required for successful host–parasite interaction is the result of ordered changes in the genetic make-up and/or gene expression in the promastigotes.

**Table 3:** List of proteins differentially expressed (≥1.5-folds) between early and late passages identified by MALDI-ToF MS/MS.

Spot Number	Assembly gap? location^∗^	Protein annotation	Accession number of protein	Protein score	Exp/Thr M.W	Exp/ Thr pI	^a^Fold change (MS/MS)	^b^Fold change Values using RNAseq
1	Yes	Constitutive major surface protease	CAC37962.1	434	68/63.8	5.9/6.84	0.66	–0.67
2	Yes	Constitutive major surface protease	CAC37962.1	393	68.5/63.8	5.7/6.84	0.45	–0.42
3	Yes	GP63, leishmanolysin	XP_001463701.2	75	69/63.8	5.6/6.84	0.05	–0.42
4	Yes	GP63, leishmanolysin	XP_001463701.2	114	40/63.8	5.4/6.84	0.1	–0.42
5	Yes	Beta tubulin	XP_003878331.1	347	55.8/49.7	5.2/4.71	6.38	0.22
6	Yes	Putative heat shock 70-related protein 1, mitochondrial precursor	XP_003877392.1	271	68.5/63.8	5.8/6.84	0.23	–0.46
8	Chr36: 1099755–1098367	Putative dihydrolipoamide acetyltransferase precursor	XP_001469441.1	382	62.4/48.62	6.4/7.02	0.44	–0.76
9	Yes	^∗∗^Activated C kinase protein, partial	ABS82039.1	103	31.2/30.63	6.7/6.63	0.66	–0.20
10	Chr32: 766497–767468	Putative heat shock protein-like protein	XP_001467884.1	308	22.5/35.49	5/4.94	0.32	–0.24
11	chr36: 2665342–2666118	Conserved hypothetical protein	XP_001469385.1	703	24/29.12	5.4/5.82	0.24	0.54
12	chr25: 350814–352157	^∗∗^Gamma tubulin	XP_003860740.1	274	22.9/49.7	5.2/4.71	0.28	–0.16
13	chr23: 9640–10320	^∗∗^Mitochondrial peroxiredoxin	AAX73294.1	314	20.6/25.34	5.4/6.43	0.27	–0.82
14	chr22: 569013–569636	i/6 autoantigen-like protein	XP_001465646.1	156	26.2/23.01	6/5.68	0.37	–0.03
15	chr36: 1493409–1494356	Hypothetical protein, conserved (MORN repeat)	XP_003865511.1	142	18.1/35.67	4.8/5.90	0.22	–0.16
16	chr35: 896773–897597	Putative RNA-binding protein	XP_003392812.1	344	22.6/30.25	6.5/7.85	0.61	–0.05
17	chr20: 601988–602383	Putative small myristoylated protein-1	XP_001465265.1	56	17.4/12.94	6.4/4.70	1.96	0.56
18	chr35: 552681–553127	^∗∗^Putative ubiquitin-conjugating enzyme E2	XP_001568232.1	46	13.1/16.65	6.3/6.08	0.43	–0.03
19	chr23: 133149–134204	Putative NADP-dependent alcohol dehydrogenase	XP_001465717.1	388	43.2/38.45	6.5/5.96	0.54	–1.48
20	chr25: 458908–460485	Putative ATPase beta subunit	XP_001466152.1	211	60.5/56.32	5.5/5.14	2.06	0.62
21	chr28: 488208–489566	^∗∗^Phenylalanine-4-hydroxylase	XP_001684410.1	71	48.8/51.48	6.2/5.93	0.37	–0.21
22	chr36: 2726362–2727795	^∗∗^Protein disulfide isomerase	XP_001469404.1	206	55.5/52.34	5.5/5.42	0.34	–0.23
23	chr25: 671470–672522	Putative pyruvate dehydrogenase E1 beta subunit	XP_001466210.1	72	45.8/37.84	5.5/5.64	0.27	–2.02
24	Yes	Alpha tubulin	XP_003873239.1	113	14.9/49.75	4.8/4.89	0.35	–0.04
25	chr21: 9660–11141	Conserved hypothetical protein	XP_001564720.1	55	23.4/52.12	5.6/6.45	0.43	–0.13
26	chr21: 413285–414697	Alpha tubulin	XP_003873239.1	68	11.3/49.75	5.6/4.89	0.36	–0.04
27	chr7: 303267–303773	Conserved hypothetical protein/putative Qa-SNARE protein	XP_003872214.1/ XP_001463282.1	59	11.5/14.35	5.2/9.15	0.52	–1.24
28	Yes	Conserved hypothetical protein	XP_001470159.1	82	33.5/24.26	5.6/5.71	0.61	–0.62
29	chr28: 460266–462242	^∗∗^Putative glucose-regulated protein 78	XP_001470161.1	207	75.2/71.94	5.5/5.05	1.55	0.04
30	Yes	^∗∗^Putative calmodulin	XP_001463554.1	50	55/70.47	5.6/4.41	0.45	–0.54
31	Yes	^∗∗^*S*-adenosylmethionine synthetase	XP_003392704.1	73	48.5/43.12	6.1/5.49	0.45	–0.08
32	Chr30:122: 5029–1226102	Conserved hypothetical protein	XP_001467184.1	115	30.2/40.83	5.9/5.32	0.11	–0.16
33	chr36: 1115280–1116995	^∗∗^Mitochondrial ATP-dependent zinc metallopeptidase, putative, metallo-peptidase, Clan MA(E), Family M41	CCM19617.1	52	19.47/72.38	5.7/8.49	0.57	–0.99
34	chr11: 473373–474023	Elongation factor 1-alpha	XP_003392396.1	112	52.36/49.12	6.8/9.03	0.61	–1.31
35	chr25: 548571–549221	Putative GTPase	XP_001463009.1	223	12.4/24.24	5.9/6.09	0.29	–0.31

*^∗^Assembly location was calculated in reference to early passages. ^∗∗^These spots were located on both gels but identified once. Exp/Thr M.W., Experimental/Theoretical molecular weight. Exp/Thr pI, Experimental/Theoretical isoelectric point. Fold change, mean upregulation or downregulation with respect to early passage. ^*a*^A value greater than 1.5 in column ‘a’ indicates up-regulation and less than 1/1.5 = 0.66 indicates down regulation. ^*b*^Negative values indicates down regulation and positive values indicate upregulation.*

An upregulated calpain like protein related to small myristoylated protein-1 (SMP-1) may also signify the struggle of late passage promastigotes to transform into infective metacyclic forms and as already mentioned in the previous section. It is also interesting to note that some calpain-like proteins, particularly those lacking the protease domain, are upregulated in the late passages while some others with protease activity are downregulated or psedogenized in the avirulent late passage parasites. Another consistently upregulated protein in the late passage was a putative ATPase beta subunit. Although there is no report in protozoans, a report linked increased acid sensitivity of bacterial pathogen to ATPase overexpression (McEntire et al., 2004). This may explain partly the non-viability of late passage parasites inside acidic host phagolysosomes. Beta tubulin was significantly up regulated in the non-infectious stage of the pathogen (Coulson et al., 1996). On the contrary alpha tubulin was mostly down regulated in the later passages. Tubulin plays a very important role in cytoskeleton formation and its expression is post-transcriptionally controlled and tightly linked to parasite transformation (Ramirez et al., 2013b). During the infection cycle, the pathogen utilizes hosts cytoskeletal machinery for pathogenicity (Gruenheid and Finlay, 2003) probably leading to less expression of these proteins.

Our study indicates that pathogenicity in *L. donovani* is a complex mechanism wherein the parasites are pre-adapted to a pathogenic lifestyle where nutritional stress and environmental sensing lead to global changes in both genes and gene expressions, ultimately leading to the production of effector molecules inside the host culminating in infection. The heteroxenic lifecycle and pleomorphic nature of the parasite adds another dimension to this process as successful transformation to metacyclic promastigotes determines virulence phenotype both in *in vivo* (other studies) in sandfly and *in vitro* (others and our study) in culture medium. It is re-established from these studies that long term cultivation of *Leishmania* promastigotes in axenic environment diminishes their adaptability toward intracellular life, and many of the factors involved in this process are markers of natural virulence attenuation. *Leishmania* have evolved several strategies to sense their immediate environment individually as well as collectively and respond to the same by controlling gene expression, and ultimately cell division and transformation. Transporter proteins are known to be involved in this pathway in other pathogens (Hlavacek et al., 2009). Continuous axenic cultivation possibly reverses this pathoadaptive response by inducing mutations in these genes which leads to loss of virulence. Further, pseudogenization of calpain like proteins involved in signal transduction and parasite transformation results in growth arrest at non-infective stages. Studies on *in vitro* cultured drug resistant and sensitive clinical isolates of *L. donovani* provided evidence that the former system has added advantage of increased metacyclogenesis in culture and higher infectivity index in host cells (Vanaerschot et al., 2010), which is also reflected in the miltefosine sensitivity assay done on the early and late passage promastigotes. Moreover, early and late passage promastigotes display differential infectivity (this work) because interaction of virulent *L. donovani* with host macrophages triggers differential transcriptional modulation in the latter as compared to avirulent strains (manuscript communicated elsewhere). A parallel gene expression study in the intracellular amastigotes also pointed toward higher expression of transporter and calcium-dependent cysteine-type endo peptidase activity genes (GO: 0005215; GO: 0004198) in more virulent forms (manuscript communicated elsewhere), strengthening the relevance of pre-adaptation in virulent parasites. The most important factor responsible for adaptive changes is the nutritional status of the immediate environment, sensing which the promastigotes undergo morphological and metabolic switch. This phenomenon is also strongly connected with transporter system in parasitic protists (Dean et al., 2014). In our genetic studies, we found domain duplication in acetyl CoA synthetase gene in the early passage promastigotes. Recent works have highlighted the importance of this enzyme in *L. donovani* infectivity owing to lipid and ergosterol biosynthesis (Soumya et al., 2017). On a broader note, this enzyme and its product acetyl CoA has been linked to global gene expression and chromatin regulation (van der Knaap and Verrijzer, 2016). This supports the theory of differential gene expression, and epigenetic changes in the virulent versus avirulent parasites, and reiterates the fact that nutrition and transcription are tightly linked. Another facet of the study relates to the importance of DNA repair and recombination activity prevalent in these parasites which promote antigenic variation suitable for intracellular adaptability and pathogenicity and needs further delving. This was indicated by differential regulation of MutS like protein at transcriptional level which has been implicated in quorum sensing independent developmental regulation in *Trypanosoma* recently (Zimmermann et al., 2017). Nevertheless, this *in vitro* cultured parasite model of gene regulation and virulence provides a basis for understanding not merely parasite adaptation to culture conditions and its genetic basis but also underlines the fact that common pathways are involved in intraspecific and intrastrain variations observed in *Leishmania*. Most of the analysis done so far in literature is based on comparison of several clinical isolates in *Leishmania*. However, comparison of various cultured passages of promastigotes are reported here.

Interestingly, in this study, we report a large number of ABC transporters undergoing structural changes in later passages, thus their inactivation may be a possible cause of loss of virulence. We have also found that in addition to several important genes such as GP63 and heat shock proteins which are already known to play major role in virulence, calpain like cysteine proteases may have significant role in loss of virulence since the genes undergo alteration and the expression levels change.

This work re-establishes the findings of other studies at transcriptomic and proteomic level to exercise caution while using serially passaged promastigotes for infectivity and therapeutic studies but also adds genetic analysis to this list. The revelation from our study that the NCBI reference strain was derived from clinical isolate which was cultured *in vitro* for multiple passages reiterates that subtle changes in the genome due to axenic cultivation may have important implications on the virulence phenotype. Moreover, similar genomic modifications may come into play when *Leishmania* infects different hosts, different vectors or even different sites of the same host which leads to differential host responses (Elso et al., 2004). Thus within-host or even within-vector selection pressure appears to be the major factor driving the modifications in the parasite which make them adaptable to their environment (Shaw, 1997), and is accompanied by no major gene losses but rather subtle polymorphisms that possibly alter the functionality of the genes. It is further evidenced from the higher expression of transporter and calpain-like proteins in visceralizing *L. donovani* strains from Sri Lanka (Zhang et al., 2014) compared to strains causing cutaneous lesions. Virulence is a function of nutritional status of the parasites and more time spent in the nutrient rich culture medium diminishes their pathoadaptive characteristics. Significant is the fact that changes in genes and gene expression occur in same/similar set of genes both in culture and in nature for virulence attenuation and cultured promastigotes can well mimic their sandfly counterparts but long-term cultures should be avoided. Changes in transporters and certain house-keeping genes can be markers of virulence. Additionally the new class of calpain peptidases are emerging as important virulence determinants along with more established metallopeptidases and cathepsin- like cysteine peptidases.

## Conclusion

The work presented here highlights the importance of newly emerging pathoadaptive factors like transporters and calpain-like proteins in *Leishmania* virulence. Our work can add to the pool of information on adaptive genomics in *L. donovani* and careful mining of these genes can be used for virulence surveillance at least throughout the Indian subcontinent. The genome sequences of early and late passage promastigotes can act as references for future genomic studies on *Leishmania*, particularly related to virulence and drug resistance. Annotated leads from this study and further annotation and functional characterization of hypothetical proteins can provide novel drug/vaccine targets for disease management.

## Author Contributions

RS and NA conceived the study. RS, R, NA, and ST designed the experiments. RS, ST, MC, SdD, and R analyzed the data. RS, ST, MC, R, and NA wrote the manuscript. RS, R, SD, and MS performed and optimized the experiments. RS, MC, SD, and ST prepared figures. RC contributed equipment and reagents. All authors critically revised and approved the manuscript.

## Conflict of Interest Statement

The authors declare that the research was conducted in the absence of any commercial or financial relationships that could be construed as a potential conflict of interest. The handling Editor declared his shared affiliation with the reviewers MB and NM.

## Footnotes

1 https://github.com/madhubioinfo/STLab-assembler
