# Supplementary material for: Genome Plasticity in Cultured Leishmania donovani: Comparison of Early and Late Passages
Source: Front Microbiol. 2018 Jul 3;9:1279. doi: 10.3389/fmicb.2018.01279 (PMC6037818; doi:10.3389/fmicb.2018.01279)
Supplement: Supplementary file 1 [file Table_1.DOCX]

| Sample | Total raw reads | No of reads after removing contaminant | Total No. of assembled transcripts | corresponding No. of genes | N50 | Total length | Average transcript length | No of id’s mapping to NCBI ref genome | GC% |
| --- | --- | --- | --- | --- | --- | --- | --- | --- | --- |
| HTI_1  (Early (2nd) passage) | R1=37710286  R2=37710286 | R1=36177703  R2=36177703 | 14579 | 11300 | 4674 | 39930360 | 2738.90 | 14439 | 59.50 |
| HTI_2  (Intermediate (11th) passage) | R1=42290498  R2=42290498 | R1=41170676  R2=41170676 | 15514 | 12314 | 4073 | 38078013 | 2454.43 | 15398 | 59.44 |
| HTI_3  (Late passage (25th)) | R1=37082434  R2=37082434 | R1=36227877  R2= 36227877 | 15447 | 12081 | 4259 | 39370999 | 2548.78 | 15323 | 59.53 |
| Merged  (HTI1_HTI2_HTI3 | HTI1 , HTI2 , HTI3 | HT1, HT2, HT3 | 28756 | 21909 | 4809 | 81169077 | 2822.68 | 28336 | 59.48 |

**Table S1.** Transcriptome assembly.

**Table S2.** Core genome completeness using BUSCO pipeline.

| Genome | COGs present | COGs absent |
| --- | --- | --- |
| HTI4 | 260 | 169 |
| HTI5 | 258 | 171 |
| NCBI | 267 | 162 |
