# Supplementary material for: Genome Plasticity in Cultured Leishmania donovani: Comparison of Early and Late Passages
Source: Front Microbiol. 2018 Jul 3;9:1279. doi: 10.3389/fmicb.2018.01279 (PMC6037818; doi:10.3389/fmicb.2018.01279)
Supplement: Supplementary file 2 [file Presentation_1.PDF]

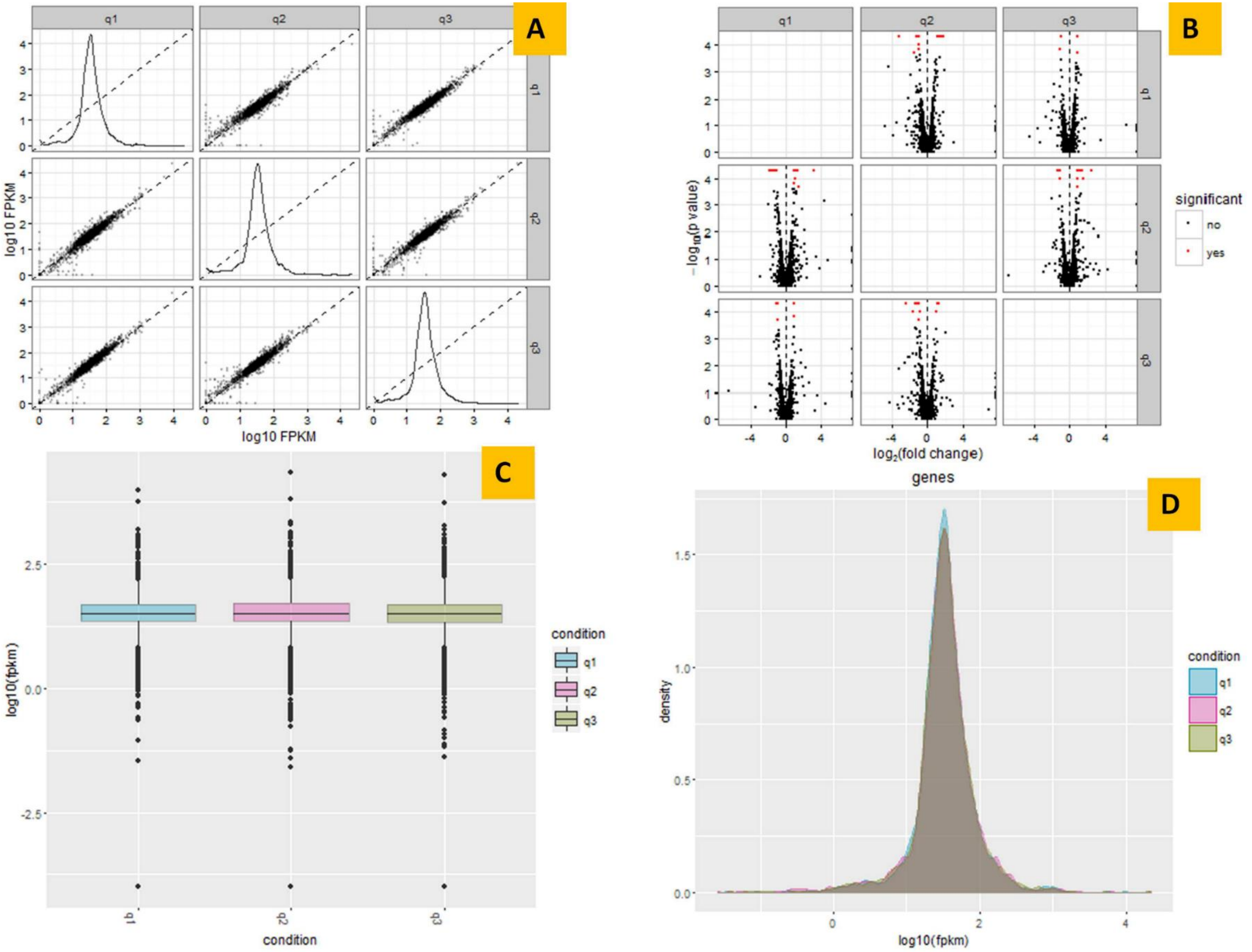

Figure S1. CummeRbund plot of transcript expression profiles. q1: Early passage, q2: Intermediate passage and q3: Late passages. (A) Pair wise comparison of transcript log10 FPKM values; density plots generated in cummeRbund represent the distribution of log10 FPKM values for that pair. (B) Volcano plot of differentially expressed genes represented as FPKM values. Red dots indicate significance in the differentially expressed genes. (C) Box plots of log10 FPKM expression values. (D) FPKM density plot overlay comparing the distribution of L. donovani transcript FPKM values between individual samples.

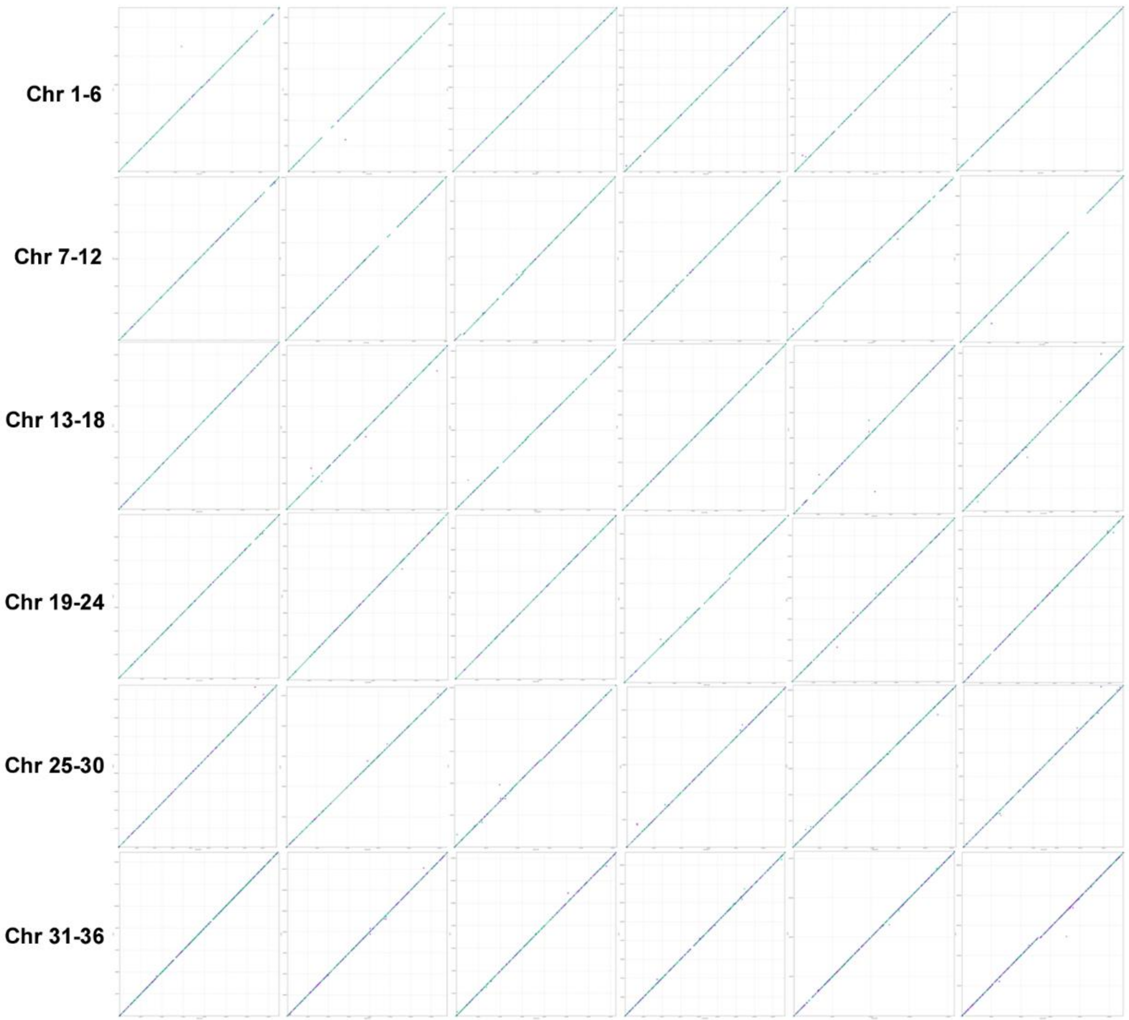

Figure S2. Whole genome chromosome wise Comparisons of early and late passages of L.donovani AG83 genomes. The x-axis denotes the chromosomes of early passage aligned with the late passage plotted on y-axis using Mummer. Purple dots from the alignment indicate the SNPs in the genomes.

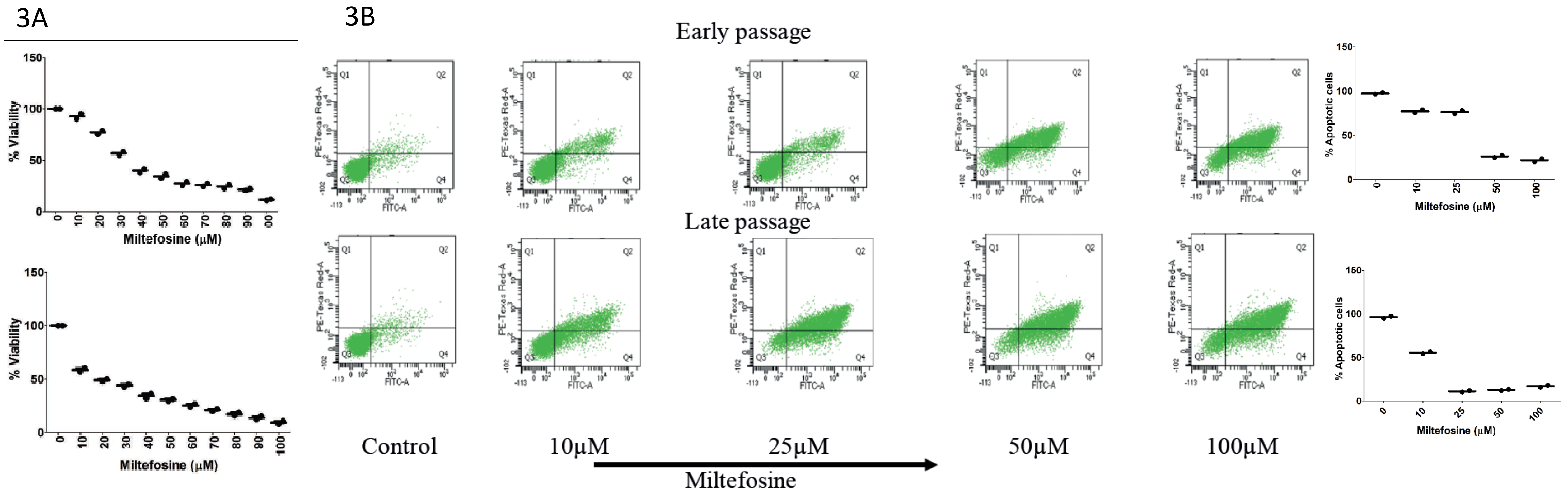

Figure S3. (A) Miltefosine sensitivity of early and late passages. 1 x10^7 promastigotes of early (upper panel) and late passages (lower panel) were incubated with or without miltefosine (doses indicated in figure) and cell viability was determined by MTT assay.(B) Apoptotic cell death was assessed in promastigotes of early and late passages by annexinV-FITC and PI staining and data was acquired on FACS LSR Fortessa. Viability data represents one experiment performed in duplicates. Representative dot plots from two independent apoptotic assays is shown and the percent apoptotic cells from two experiments is plotted against increasing drug concentration.

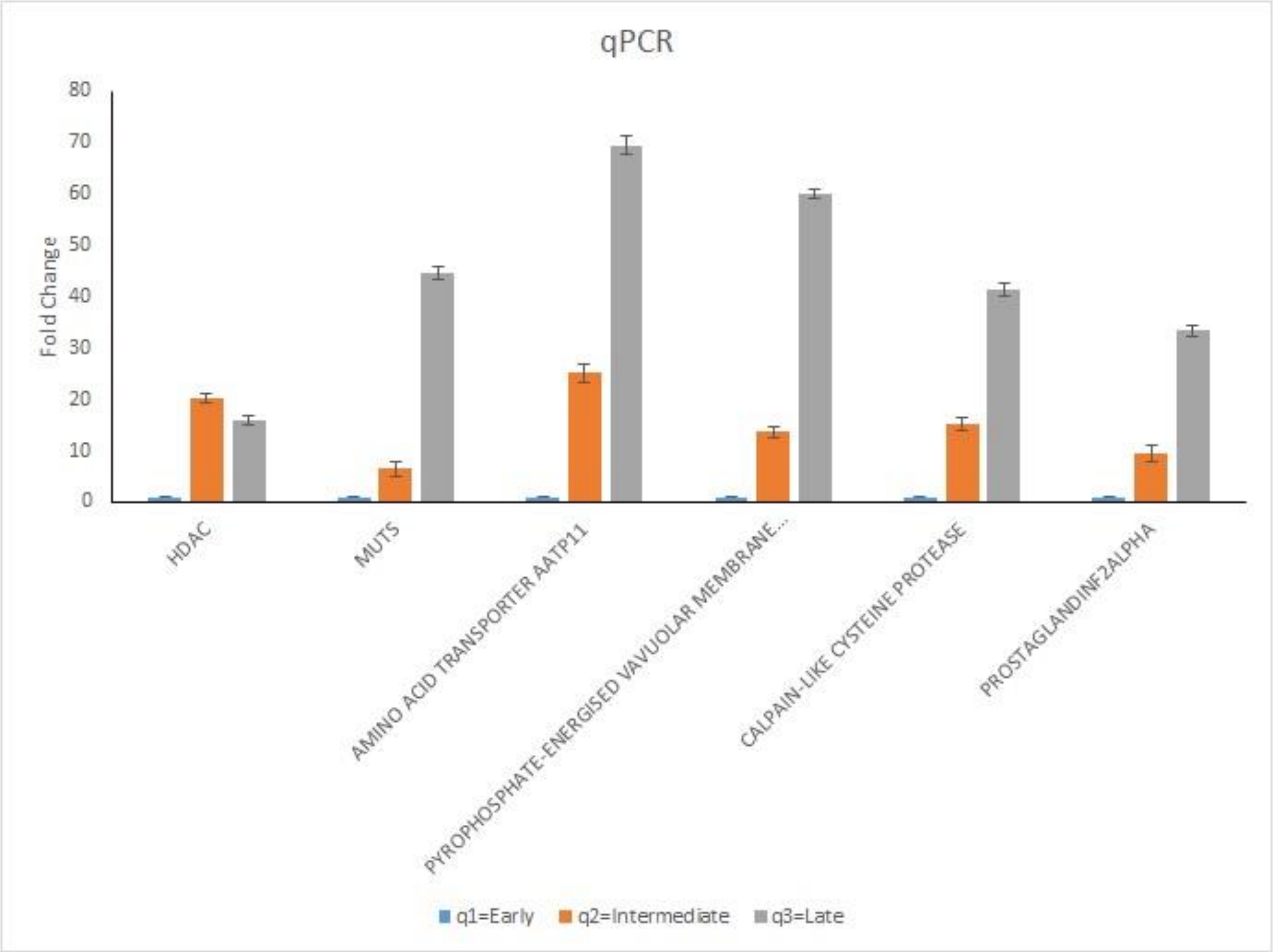

Figure S4. Comparative gene expression of transcripts. The mRNA expression of few genes differentially expressed between early, intermediate and late passages in RNAseq were confirmed by qPCR. Data is represented as fold change ± S.E. of 3 independent experiments.

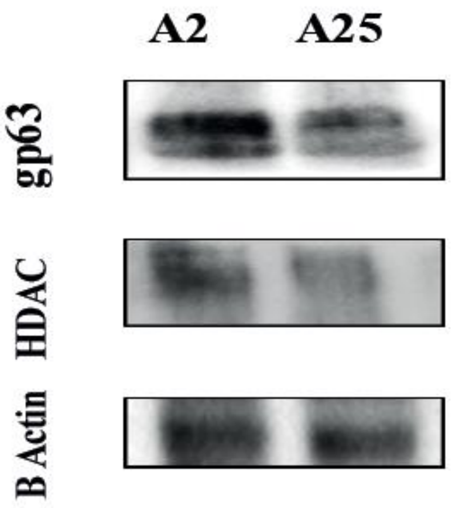

Figure S5. Differential protein expression in early and late passage promastigotes. Total cell lysates prepared from 1 x10^7 promastigotes of early and late passages were run on SDS-PAGE and incubated with anti-gp63 or anti-HDAC antibody. The images were developed and analysed on Bio-Rad XR gel doc.

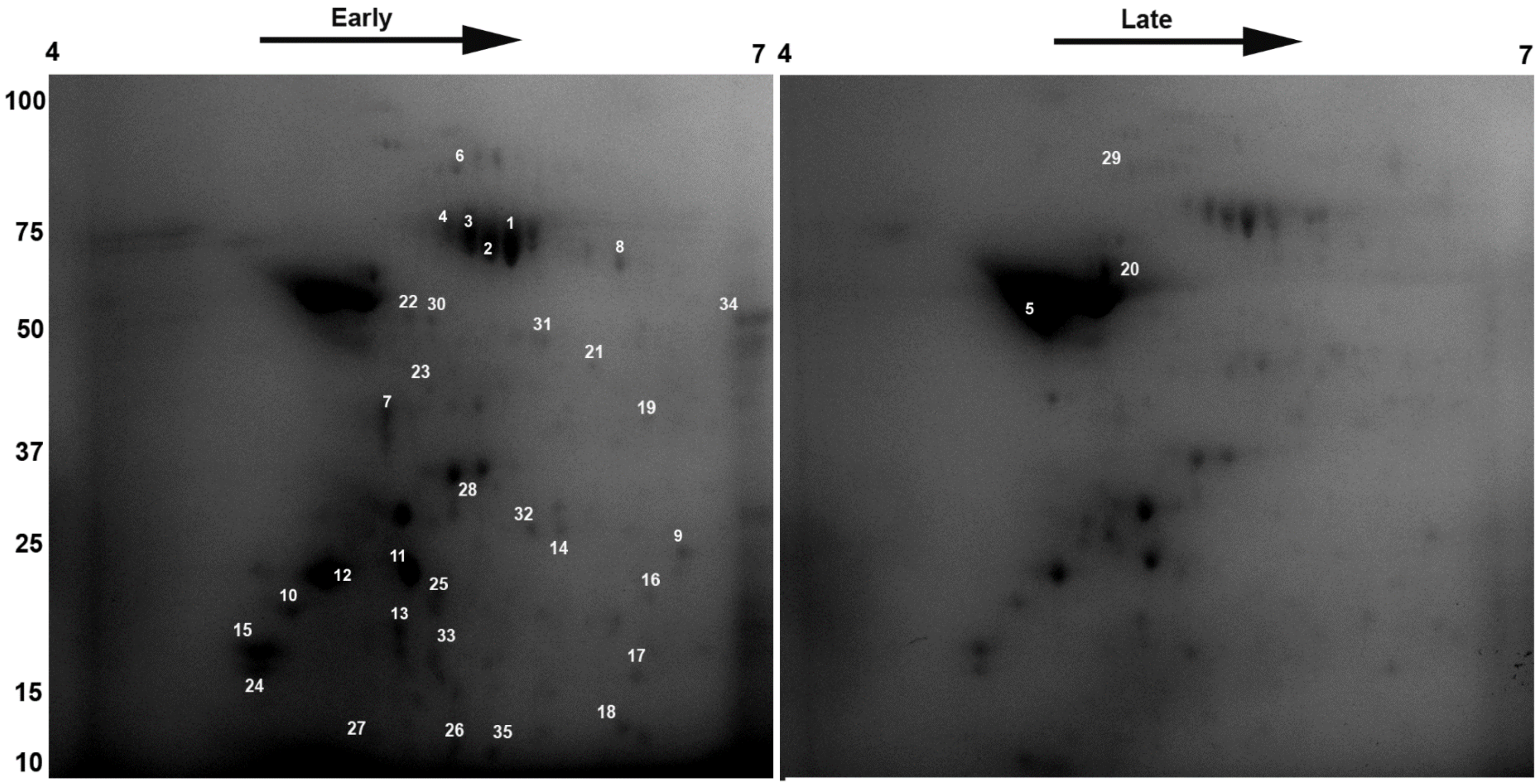

Figure S6. Two-dimensional profiles of cultures from Leishmania donovani early and late passages. The 2-DE gels were obtained using crude membrane extracts of L. donovani AG83 of early (2nd – 5th passage) and late (22nd – 25th passage) passages.500μg of each extractwas run on 2-DE (first dimension: IEF pH range 4–7; second dimension: 12% SDS-PAGE) and stained with colloidal Coomassie Brilliant Blue G-250. One representative gel of each sample from two independent studies is shown here. The spot numbers are marked on the gels having higher expression of a particular protein.
